# Skimming Digits: Neuromorphic Classification of Spike-Encoded Images

**DOI:** 10.3389/fnins.2016.00184

**Published:** 2016-04-28

**Authors:** Gregory K. Cohen, Garrick Orchard, Sio-Hoi Leng, Jonathan Tapson, Ryad B. Benosman, André van Schaik

**Affiliations:** ^1^Biomedical Engineering and Neuroscience, The MARCS Institute, Western Sydney UniversitySydney, NSW, Australia; ^2^Natural Vision and Computation Team, Vision Institute, University Pierre and Marie Curie-Centre National de la Recherche ScientifiqueParis, France; ^3^Temasek Labs (TLAB), National University of SingaporeSingapore, Singapore; ^4^Neuromorphic Engineering and Robotics, Singapore Institute for Neurotechnology (SINAPSE), National University of SingaporeSingapore, Singapore

**Keywords:** N-MNIST, object classification, OPIUM, SKIM, multi-class

## Abstract

The growing demands placed upon the field of computer vision have renewed the focus on alternative visual scene representations and processing paradigms. Silicon retinea provide an alternative means of imaging the visual environment, and produce frame-free spatio-temporal data. This paper presents an investigation into event-based digit classification using N-MNIST, a neuromorphic dataset created with a silicon retina, and the Synaptic Kernel Inverse Method (SKIM), a learning method based on principles of dendritic computation. As this work represents the first large-scale and multi-class classification task performed using the SKIM network, it explores different training patterns and output determination methods necessary to extend the original SKIM method to support multi-class problems. Making use of SKIM networks applied to real-world datasets, implementing the largest hidden layer sizes and simultaneously training the largest number of output neurons, the classification system achieved a best-case accuracy of 92.87% for a network containing 10,000 hidden layer neurons. These results represent the highest accuracies achieved against the dataset to date and serve to validate the application of the SKIM method to event-based visual classification tasks. Additionally, the study found that using a square pulse as the supervisory training signal produced the highest accuracy for most output determination methods, but the results also demonstrate that an exponential pattern is better suited to hardware implementations as it makes use of the simplest output determination method based on the maximum value.

## 1. Introduction

The need for visual sensing in commercial technologies has experienced a rapid increase, often driven by the growing field of autonomous devices. In many cases, the growth and applicability of such systems are restricted by the speed, latency, and power consumption of the current algorithms and hardware used in computer vision. Autonomous systems represent one of the most challenging tasks for a computer vision system with performance limited by strict power budgets and requiring fast and accurate processing with a high cost of failure.

Visual sensing is also an important task in the biological realm, and is heavily relied upon by many different organisms. Sighted animals use vision to effectively sense and react to the surrounding environment, often with extreme restrictions placed on latency, speed, and energy consumption. These biological systems overcome many of the problems faced by conventional computer vision, and do so in a manner that is many orders of magnitude more efficient in terms of power consumption.

Although it is extremely difficult to model and reproduce the methods used by biology in order to sense and process visual information, there is merit in examining the overarching mechanisms and paradigms used in biology to design systems that are biology-inspired. This paper unites two such systems, a biology-inspired silicon retina and a learning method based on the notion of dendritic computation. The methods presented in this work explores a new approach to visual sensing, specifically for the purposes of classification. It is important to stress that the systems presented in this paper are not biologically realistic, but rather are inspired by biological approaches to sensing and computation.

This paper begins by introducing a number of recent advances in artificial visual sensing and computation, with a focus on systems that make use of silicon retinas. This is followed by a short description of the Synaptic Kernel Inverse Method (SKIM), which forms the learning mechanism used in this work. This is followed by a discussion of the classification methodology and the means by which SKIM is extended to process visual information. The results of the experiments are then presented along with a discussion and conclusion.

### 1.1. Recent advances

The recent advances made in artificial visual sensing have occurred primarily in three areas. The first deals with the process of capturing visual information from scenes in an efficient manner using neuromorphic devices called *silicon retinae* which preserve accurate timing of log-intensity changes in the scene. This accurate timing has been shown to carry additional information (Akolkar et al., 2015). Inspired by their biological counterparts, these devices use analog circuits at each photosensitive element to perform computation and compression, and transmit this information in a spike-based manner. A full treatment and in-depth review of these *silicon retinae* can be found in Posch et al. (2014) and the device used in this paper makes use of the Asynchronous Time-Based Imaging Sensor (ATIS) described in Posch et al. (2011).

There have also been a number of significant advances in the design and development of large-scale biology-inspired spiking neural hardware, which forms the second area of advancement. These hardware devices compute in a power efficient manner inspired by neurons in the brain and can be used to process the captured visual signals from neuromorphic devices such as *silicon retinae*. Prominent examples of these systems include SpiNNaker (Painkras et al., 2013), Neurogrid (Benjamin et al., 2014), and the TrueNorth processor from IBM (Merolla et al., 2014), which is capable of implementing one million neurons and 256 million synapses in real time and is reported to consumption of less than 100 mW.

Finally, the third area of advances concern the design of Spiking Neural Network (SNN) algorithms, which make use of the differing paradigm of hardware and sensors to extract information and provide computation. Examples of these systems include the SKIM presented in Tapson et al. (2013), the HFIRST algorithm found in Orchard et al. (2015b), as well as multi-layer deep learning approaches as explored in (Pérez-Carrasco et al., 2013; O'Connor et al., 2013).

This paper presents an implementation of SKIM networks to the event-based output of a *silicon retinae* in order to perform a large-scale classification task. Such steps are critical in proving the viability and efficacy of Spiking Neural Network algorithms. Although the algorithm presented is a software simulation, the design of the system in an event-based manner naturally lends itself to implementation on the emerging spike-based computational hardware.

### 1.2. Synaptic kernel inverse method

The SKIM, proposed and outlined in Tapson et al. (2013), is a neural synthesis technique which produces networks of neurons and synapses that are capable of implementing arbitrary functions on spike-based inputs. The network generally contains a single input neuron for each input channel, and a single neuron for each desired output channel. The conventional fan-out to a higher dimensional space, present in most Linear Solutions to Higher Dimensional Interlayer (LSHDI) network systems (as introduced and described in Tapson et al., 2013) and usually implemented through a hidden layer of neurons, is replaced with multiple synaptic connections, which are shared between output neurons.

SKIM differs from other LSHDI systems, such as the Extreme Learning Machine (ELM), in that it is specifically designed to learn spike-timing dependent signals. It therefore bears a closer resemblance to synthesis methods such as the Neural Engineering Framework (NEF), which is also capable of spike-based input-output relationships (Eliasmith and Anderson, 2004). SKIM differs from the NEF in that it does not rely on rate-encoded signals, and rather relies on both the spatial and temporal information in the incoming spike-trains.

**Figure 1:**
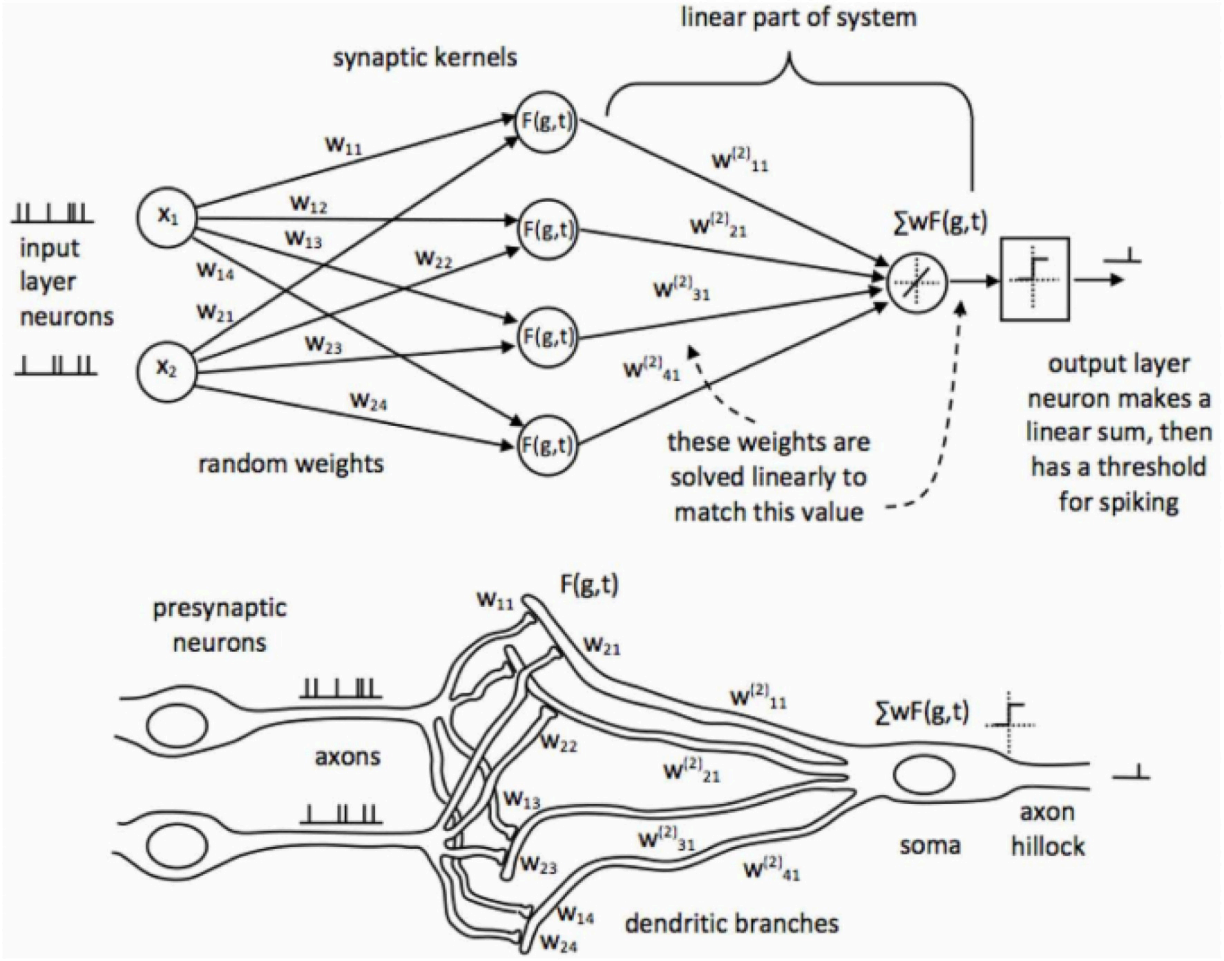
**Topology of the Synaptic Kernel Inverse Method (SKIM) as shown in Tapson et al. (2013)**. Each input layer neuron (left) will be an input from a separate pixel. At initialization, the static random weights and synaptic kernels are randomly assigned and they remain fixed throughout the learning process. During learning, the output weights in the linear part of the system are solved to minimize the error between the system output and the desired output.

SKIM is based on a biologically plausible network structure modeled on the synaptic connections between neurons. An overview of the SKIM network is shown in Figure 1. In the SKIM, input neurons are considered analogous to pre-synaptic neurons and input event streams are projected to a layer of synapses through a set of random weights. Each weight is representative of a specific dendritic branch leading toward a synapse. These synapses implement non-linear responses to the received current from the pre-synaptic dendritic branches through the use of non-linear kernels, such as exponentials or decaying-alpha functions. It is these kernel functions that provide the SKIM with the ability to respond to temporal information in the input as they convert discrete incoming spikes into a continuous value.

The outputs of these synapses proceed down the post-synaptic dendritic branches, which connect to the soma of the output neurons. The dendritic branches sum the currents from the synapses at the soma of the output neuron, causing it to fire if the soma potential exceeds a specified threshold. It is the properties of these post-synaptic dendritic branches which are analytically calculated in the SKIM method as they are analogous to the linear output weights in other similar systems.

The linear output weights are iteratively calculated using the Online Pseudo-inverse Update Method (OPIUM; van Schaik and Tapson, 2015), which allows for the iterative calculation of an analytical solution for the weights.

### 1.3. Dataset

This work makes use of the N-MNIST spiking neuromorphic dataset presented by Orchard et al. (2015a), which was created specifically to provide a benchmark for neuromorphic vision algorithms. Additionally, it serves to provide a spike-based analog to the corresponding MNIST dataset, first presented by LeCun et al. (1998), and which provides an important and well-understood benchmark for the computer vision community.

The conversion process involved the use of an ATIS camera attached to a pan/tilt mount and positioned in front of an LCD screen. The converted recordings for each image in the datasets consists of the event-based output from the camera as the pan/tilt mechanism through across three saccade-like motions described in Orchard et al. (2015a). The output of each consists of a set of events in the Address-Event Representation (AER), as described in Boahen (2000).

The N-MNIST dataset contains 60,000 training digits and 10,000 testing digits with an approximately equal number of samples of handwritten digits in the range of 0–9. Each image consists of a single, centered digit on a white background, and was encoded to an event stream with a spatial resolution of 34 × 34 pixels. Although the original MNIST digits are 28 × 28 pixels in size, in order to account for the saccade motion, a 34 × 34 pixel resolution was required.

### 1.4. Contributions

The work presented in this paper makes a number of contributions to event-based vision processing and the SKIM algorithm. It demonstrates the efficacy and applicability of a spike-based approach to classification using real-world event-based data. Through the applications of the methods described in this paper, this paper presents the highest accuracy achieved on the N-MNIST dataset to date.

This work also extends the original SKIM algorithm to multi-class problems, exploring the different methods of determining the winning output class in such systems. Four different output determination methods are described and discussed. Additionally, the work also explores various training patterns for use in SKIM networks, and fully explores and characterizes the resulting accuracy. Finally, these two aspects are combined to give insight into the methods of applying algorithms such as SKIM to event-based visual classification systems.

## 2. Materials and methods

Applying the SKIM algorithm to a classification task with the scale of the N-MNIST dataset requires a number of modifications to the underlying SKIM algorithm and a complete software framework. The framework needs to manage the state, implement the neural layers, collate the outputs and manage the training and testing regime.

Prior works have explored the theoretical performance of SKIM networks using pre-determined patterns with varying levels of noise and jitter. The authors applied the technique to the Mus Silica dataset in the original SKIM paper Tapson et al. (2013) and then later applied the SKIM to a real-world separation problem Tapson et al. (2015). Others have used the algorithm to determine angle and direction in biological motion estimation, and in gait detection Lee et al. (2014).

The application of SKIM to the N-MNIST dataset requires networks with an order of magnitude more input neurons and synapses than previous SKIM networks. In addition, the majority of the applications to date have formulated their outputs as binary classification tasks, whereas the N-MNIST is inherently a 10-class problem.

The training methods used for the prior SKIM implementations have, to date, created a single pattern consisting of all the training data, accompanied by a supervisory learning pattern of equal duration. The input pattern is a spatio-temporal pattern representing spikes arriving at the input nodes of the SKIM network, and the supervisory pattern contains the desired output spikes from the output neurons.

When dealing with the N-MNIST dataset, the direct application of the above method is not feasible due to the size and number of the patterns. Instead, the SKIM implementation used in this work trains each pattern in a stand-alone fashion, preserving the random weights, the configuration of the hidden layer nodes, the inverse correlation matrix and the linear output weights between training sequences. This allows the network to train on any number of training samples as it is no longer bound by the available memory.

When training the SKIM networks with the N-MNIST dataset, the digit sequences were extracted from the dataset in a random order. The training order therefore contained a randomly interleaved set of all output classes, and each sequence was only used once for training. The random training order was preserved when running multiple trials of the same network configuration. A random testing order was also used in this work, although it is not required as no updates to the network are performed.

The temporal resolution of each event in the N-MNIST dataset is reduced from the order of microseconds to milliseconds with minimal impact on number or nature of the events. This is due to the slow movement of the camera relative to the rate at which events are time-stepped. In addition, the camera biases were not configured for high-speed acquisition but rather to reduce noise and maximize the balance between ON and OFF events. This is an important step as it allows the SKIM algorithm to simulate millisecond time-steps, instead of microsecond ones, which dramatically increases the speed of computation.

Each training and testing sequence in the dataset consists of a stream of AER events and a label indicating the digit class to which it belongs. The AER events generated from the ATIS camera have the following form:

(1) $$\begin{array}{l} \text{e}={\left[x,y,t,p\right]}^{T} \end{array}$$

In the above equation, **u** = (*x, y*) denotes the spatial location of the pixel generating the event, *t* contains the value of the internal time-stamping counter on the ATIS camera at the moment at which the camera circuitry receives the event from the physical sensor and *p* ∈ [−1, 1] denotes the polarity, indicating whether it was an ON event or an OFF event. The SKIM network cannot operate on the event stream directly, and a conversion to a fully specified spatio-temporal pattern in which rows ascribe input channels and columns denote time-steps is necessary. We can denote such a spatio-temporal pattern as *I* such that *I*(*c*, δ*t*) denotes the dendritic current on channel *c* at the time step denoted by δ*t*.

The spatial information contained in **u** = (*x, y*)^*T*^ is inherently lost when applied to the SKIM network as the synaptic non-linearity discards the spatial location of channels. Therefore, any transformation that consistently maps ℝ^2^ → ℝ is a suitable candidate for the conversion of spatial locations to input channels for SKIM. This only hold true if there is no interaction between input channels. Operations such as spatial down-sampling can invalidate this condition depending on the implementation. When down-sampling spatially, the order of channels becomes significant as it dictates to which pixel (and then subsequent channel) the information from a region of pixels aggregates. All the experiments performed on the N-MNIST dataset made use of the simple mapping operation shown in Equation (2).

(2) $$\begin{array}{l} c=\left\lfloor \frac{34\times y}{\beta }\right\rfloor +\left\lfloor \frac{x}{\beta }\right\rfloor \end{array}$$

In the above equation, the constant value β represents the spatial down-sampling factor applied to the pattern and ⌊*n*⌋ operation represents the floor function applied to *n*. The down-sampling factor operates on both the *x* and the *y* coordinates, effectively reducing the number of channels by a factor of β^2^. As no down-sampling was used in this work, β = 1 for all experiments.

The value of 34 derives from the pixel dimensions of the N-MNIST digits, as opposed to the 28 × 28 pixel images from the original MNIST dataset. Down-sampling the temporal information is far simpler as *t* is a monotonically increasing single-valued variable, requiring only a division and flooring operation to quantize it into the appropriate time step as shown in Equation (3). This equation is used to reduce the resolution from microseconds to milliseconds.

(3) $$\begin{array}{l} \delta t=\left\lfloor \frac{t}{\alpha }\right\rfloor \end{array}$$

Given the temporal reduction from microseconds to milliseconds, α = 1000 for the purposes of the networks presented in this work. Therefore, for each incoming event *e*, the effect on the spatio-temporal input pattern for SKIM is as follows:

(4) $$\begin{array}{l} I\left(c,\delta t\right)\to I\left(c,\delta t\right)+p \end{array}$$

The above operation demonstrates that the effects of multiple events accumulate when mapped to the same channel and time-step, and that their polarity dictates the nature of their contribution. It is also important to remember that the value of *t* is always monotonically increasing, allowing the iterative construction of the spatio-temporal pattern, and allows processing to begin for a time step once the time value for the next event exceeds it.

The output of a SKIM network is a continuous value for each output class representing the soma potential at the output neuron. In the original SKIM implementation by Tapson et al. (2013), the application of a fixed threshold converted the continuous value into a binary spike, allowing the creation of an output spatio-temporal pattern. The nature of this output spatio-temporal pattern retains the same temporal resolution, namely the same number of equally sized time-steps, with the rows changed to represent the output of each output neuron.

As the training update in a SKIM network requires a measure of error between the network output and the desired output, it follows that the format for the learning sequence must adhere to the same format as the network output. We can therefore define the output pattern *O* such that *O*(*n*, δ*t*) represents the real-valued potential at output neuron *n* at time δ*t*. Therefore, for every δ*t*, the input pattern *I* contains the instantaneous input values for all *c* channels, and the training pattern *O* contains all the desired output values for each output neuron *n*.

The analysis of the N-MNIST dataset presented in Orchard et al. (2015a) shows that a pattern length of 315 ms is sufficient to encode every pattern in both the training and testing set. Appending an additional 45 ms onto the pattern allows the last events to have an effect on the learning system, resulting in a total pattern length of 360 ms. It is within this additional 45 ms that both the training and recall occur.

### 2.1. Training patterns

In theory, a SKIM network should require only an output spike as a supervisory training pattern. In reality, a single spike (i.e., a pulse with a duration of a single time step) does not produce an error signal with enough magnitude or duration to allow rapid learning. It is possible to train with a short duration pulse, but it requires multiple presentations of each digit and does not reliably converge. In place of a single spike, using a training pattern of a longer duration produces a better result without the need for multiple presentations of the training sequence. Having a pattern that spans multiple time-steps also allows the use of different training patterns, which can have a significant impact on both the training itself and the most appropriate method of determining the output class.

**Figure 2:**
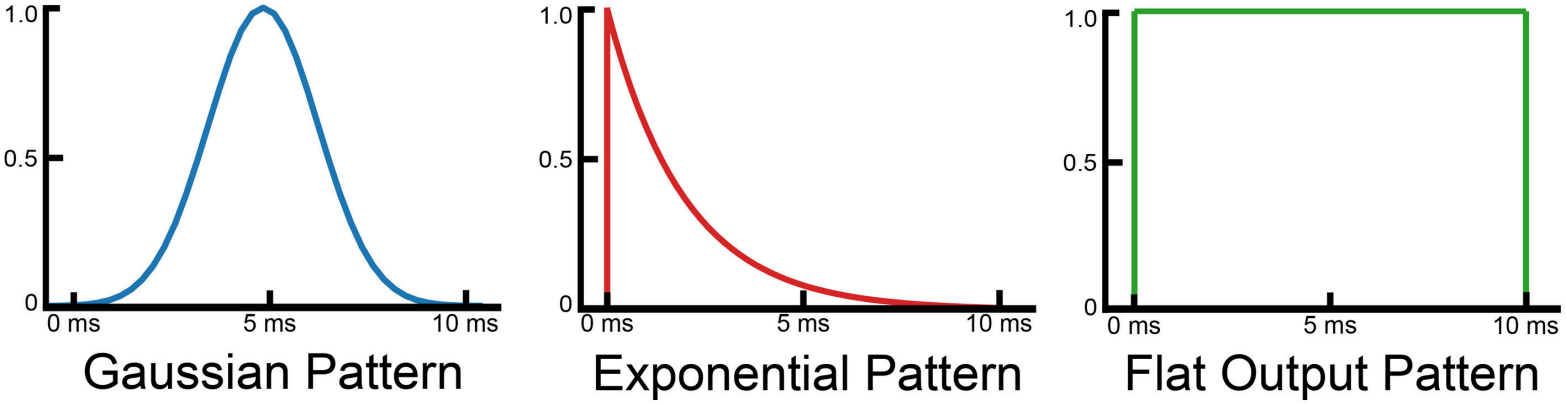
**Diagram showing different training patterns used to train the SKIM network**.

Figure 2 shows three different training patterns that produce good results with the SKIM network. The flat output pattern is the logical extension of the single output spike, but has two sharp discontinuities on each end. The Gaussian pattern represents the opposite approach, and exhibits a smooth (although discretized) curve which peaks during the middle of the output pattern. The exponential pattern represents the combination of the two approaches, and maintains the initial discontinuity but gradually decreases so as to exhibit a smooth return to zero.

To evaluate the performance of these training patterns, full tests against the N-MNIST datasets were performed. Each network contained 1000 hidden layer neurons, and made use of the full training set. Sixty trials of each experiment were performed, with only the random weights varying between trials, and the average error rate and standard deviation reported.

### 2.2. Output determination methods

All prior work with the SKIM algorithm was always limited to training a single output neuron and employed a threshold to generate output spikes from the soma potential of output neurons. If trained with the same random weights and hidden layer configuration, it is possible to independently train and then combine multiple output neurons (and their associated thresholds) to implement a multi-class classifier. As the outputs are already spikes, it is possible to use existing spike-based techniques such as first-to-spike and a winner-take-all approaches to selecting an output class. Unfortunately, due to the need for fine-tuning and determining thresholds this approach does not scale well when dealing with datasets such as N-MNIST, and there exists a need for a more robust and automated means of output determination.

In practize, multi-class problems constructed from independently trained output neurons suffer from the need for individual and specific thresholds for each output class, or the use of a global threshold which is often sub-optimal, as the ranges and characteristics of the output neurons may differ. This introduces additional parameters into the classification methodology, which is difficult to empirically determine given the size of the datasets and the time required to train on them.

In response to this issue, the approaches detailed in this section all serve to remove the need for fixed thresholds, and replace them with a comparison between output neurons directly. For this approach to work, the outputs must therefore be relative in magnitude, which requires the simultaneous training of all the output classes. Although the OPIUM method underpinning SKIM does include a normalization step, the range of the linear weights can vary from class to class when training individually, and prevents the direct comparison of output class values. When training all outputs simultaneously with SKIM (underpinned with OPIUM), the normalization applies to all output weights, keeping them relative in magnitude to one another.

This paper proposes and investigates four approaches to determining the output in a multi-class problem using SKIM, primarily applied to the N-MNIST dataset and also applicable to the multi-class classification problems in the N-Caltech101 dataset introduced in Orchard et al. (2015a). Each method utilizes the real-valued output from each of the output neurons during the section of the pattern in which the supervisory learning signal is expected to be present, avoiding the need for explicit thresholding.

**Figure 3:**
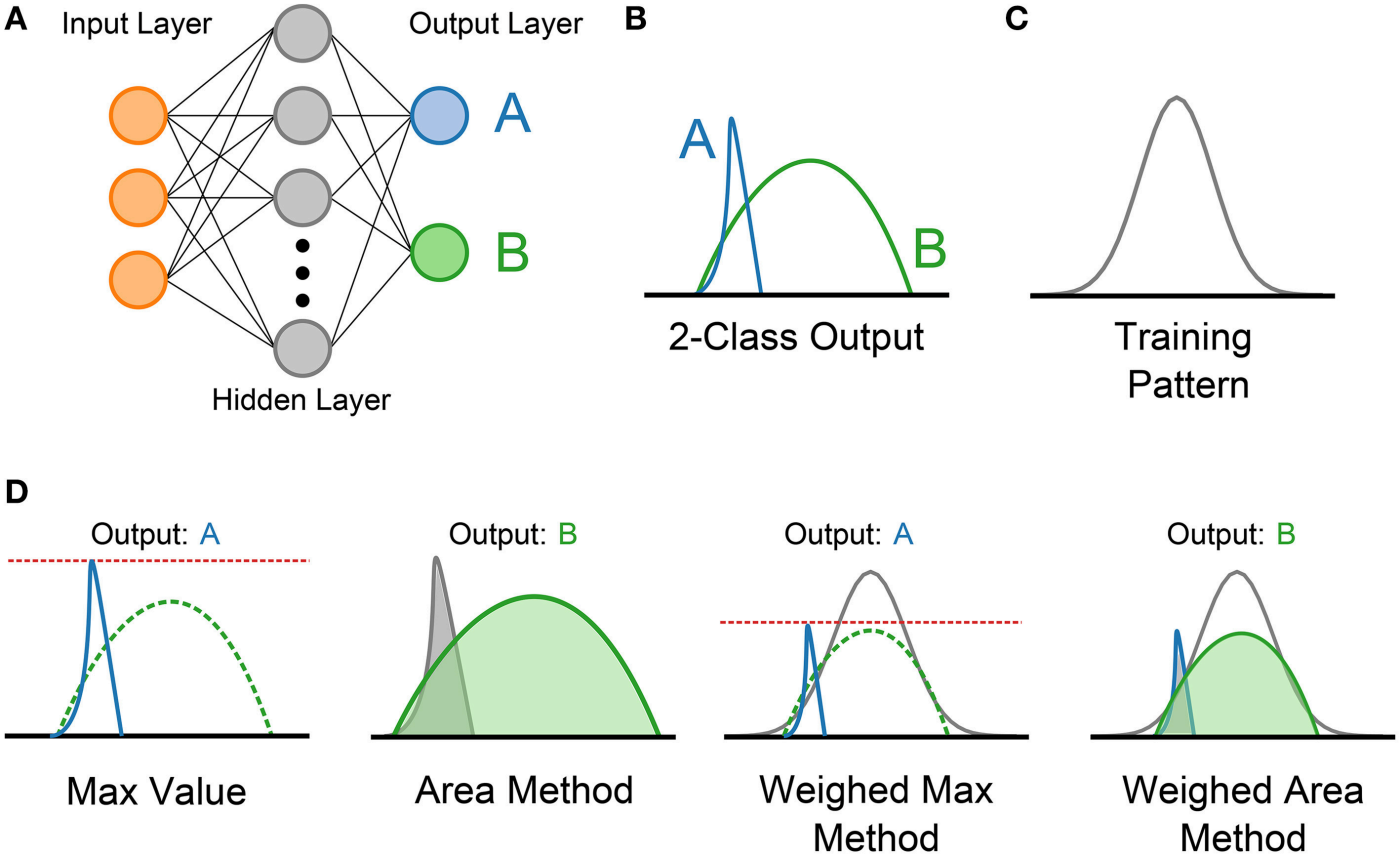
**Diagram showing the four output determination methods evaluated for use in multi-class SKIM problems**. **(A)** An example SKIM network with two simultaneously trained output neurons coding for two classes; (A,B). **(B)** Example outputs from the two output neurons during the period in which the training pattern occurs. In this example, the training utilized is the Gaussian training pattern shown in **(C)**. Diagrams showing the manner of calculating the four output selection methods are presented in **(D)**, and include a label showing the theoretical winning class in each case. Note that for the Weighted Sum and Weighted Area methods, the training pattern is also shown.

Figure 3 demonstrates the four methods used. The first approach is the Max Method, and simply takes the output class that achieves the maximum value during the output period. This maximum in the SKIM output does not necessary have to correspond with the intended location of the maximum in the training signal, but simply represents the maximum of any output class during the output phase. The second approach calculates the area under each curve, and selects the output class with the highest overall area. This is analogous to integrating the incoming values, and picking the highest value. It is important to note that the output can be either positive or negative, and any areas arising from portions of the curves below zero are negative in value and require subtracting from the total positive area.

The third and fourth methods exploit knowledge about the training pattern, and attempt to weight the outputs accordingly before applying the same techniques used in the first two methods. This has no effect when using a square training pulse, but has a significant effect when using a Gaussian or exponential training sequence (as the flat pattern results in a uniform pattern as shown in Figure 2). The third method weights the outputs proportionally to the training pattern, and then finds the maximum value. This method, dubbed the Weighted Max method, places emphasis on the portions of the output range where a high value is expected. The fourth method, referred to as the Weighted Area method, weights the output values using the input pattern and then calculates the areas under the curve and selects the highest output.

## 3. Results

The results for this paper are divided among four sections. The first section presents the classification results achieved when applying the SKIM learning method to the N-MNIST dataset. These results serve as benchmark results for SKIM networks of varying sized hidden layer networks and achieve the highest reported accuracy to date.

The second section presents the results of a statistical investigation into the normality of the distribution of results for a SKIM network. The outcome of this section is important as it provides a robust underpinning for the results presented in the next two sections, which explore the effects of the training patterns and output determination methods presented in Sections 2.1 and 2.2.

### 3.1. Classification results

As N-MNIST is a new dataset, the results presented in this paper serve to supplement the initial classification benchmarks presented in Orchard et al. (2015a). The statistical classifiers presented in that work attempted to set a theoretical lower bound on performance, and these measures are important to understanding the nature of the classification problem but additional value is also gained from applying existing and state-of-the-art spike-based classification techniques to the classification problem.

The comparison results presented in this paper represent a detailed application of the SKIM classification network to this dataset, and present the highest classification accuracy achieved on the N-MNIST dataset to date.

**Figure 4:**
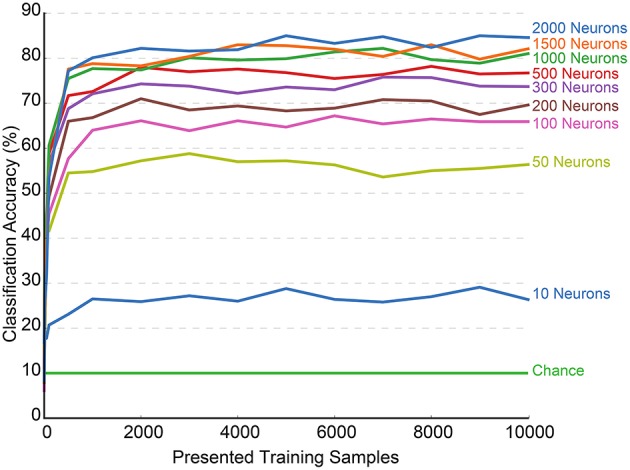
**Training accuracy over the first 10,000 presented samples for N-MNIST using SKIM for increasing numbers of hidden layer neurons**. Each configuration of hidden layer neurons was trained sequentially through a randomly shuffled training sequence, and tested against the testing dataset at increments of 1000 hidden layer neurons. Each test was performed independently of the training, and no learning took place during the testing phase. Also shown on the graph are the accuracies due to chance, which is 10% for the 10-class classification task. The final results shown on the right represent the full training accuracy tested against the full 10,000 training samples whilst intermediate points on the curve were calculated over a randomly drawn subset of 2000 testing samples.

Figure 4 presents a plot of classification accuracy (in terms of percentage of digits correctly identified) as a function of the number of training samples presented. Each curve represents the results of a network with a different hidden layer size tested against the test dataset at regular intervals during the training process. The final accuracies obtained for these networks are presented in Table 1.

**Table 1:** **Accuracy for the N-MNIST dataset after 10,000 and 60,000 training samples for different hidden layer sizes**.

**Hidden layer size**	**10,000 samples (%)**	**60,000 samples (%)**
10	26.36	26.42
100	65.92	65.79
200	69.62	70.11
300	73.70	74.07
500	76.73	76.36
1000	81.03	81.51
1500	82.10	82.31
2000	83.44	83.96

The testing phase occurred separately from the learning process, and the results were never included in the training or in any update mechanism. The plot displays only the first 10,000 samples as the network performance stabilizes and remains constant after that point. Both training and testing orders were always random.

It is interesting to note that a network trained with only 10 output neurons achieves a performance of ~26.36% at 10,000 samples, which is almost exactly the performance of 26.52% yielded by the statistical classifier trained on the number of events presented in Orchard et al. (2015a). This suggests that the network requires only 10 output neurons to learn and respond to the event counts.

The figure also demonstrates the quick convergence of the network, with the accuracy stabilizing within 2000 samples in almost every case, and often much earlier. There were no problems resulting from over-fitting, and the accuracy remained constant through the full 60,0000 training presentations. This is significant, given the iterative nature of the training process and proves the viability of using the system in an online manner.

Table 2 provides the results of fully trained SKIM networks with increasingly large number of hidden layer neurons. Whereas, the 2000 neuron network achieved an accuracy of 83.96% after training on the complete set, the network with 10,000 neurons achieved an overall accuracy of 92.87%. Due to the size and computational time required to simulate these networks, only one trial of each experiment could be performed.

**Table 2:** **Results for classification of N-MNIST with larger hidden layer sizes**.

**Hidden Layer Size**									
1000	2000	3000	4000	5000	6000	7000	8000	9000	10000
81.51%	83.96%	85.6%	85.1%	86.6%	86.3%	88.6%	90.22%	91.56%	92.87%

*This table shows results for SKIM networks with large numbers of hidden layer neurons and trained on the full 60,000 training samples. Due to the time taken to train these large networks, only a single trial of each was performed*.

### 3.2. Error analysis of the SKIM network

As the networks used in this work make use of either random weights or random training orders, it is important to conduct multiple trials of each experiment to fully characterize the networks and their performance. The results for classification tasks are often provided as either a mean accuracy, or a mean accuracy and standard deviation, and is a common practize within the machine learning community.

However, results presented in such a manner only fully characterize the error distribution when the errors are normally distributed, and this is often an implied assumption when stating results in such a fashion. This section explores and characterizes the nature of the errors arising from a typical SKIM experiment, and attempts to validate this assumption for a typical SKIM network. All statistics reported use either the standard *t*-test or the paired *t*-test as appropriate.

The network chosen to characterize was the SKIM implementation with 1000 hidden layer neurons. This same configuration was also used to explore the effects of training patterns and output determination methods as this network represents a good balance between accuracy and training time, making it well suited to experiments that require multiple trials.

Testing included the two variations of this network, making use of the Gaussian and Exponential training patterns. The characterization involved 51 full tests with all 60,000 training samples on each network, with only the random weight matrix and training order varying from trial to trial.

The networks with the Gaussian and Exponential patterns received the same random weights for each trial, and the Area method of the output determination methods run on the same network output.

**Figure 5:**
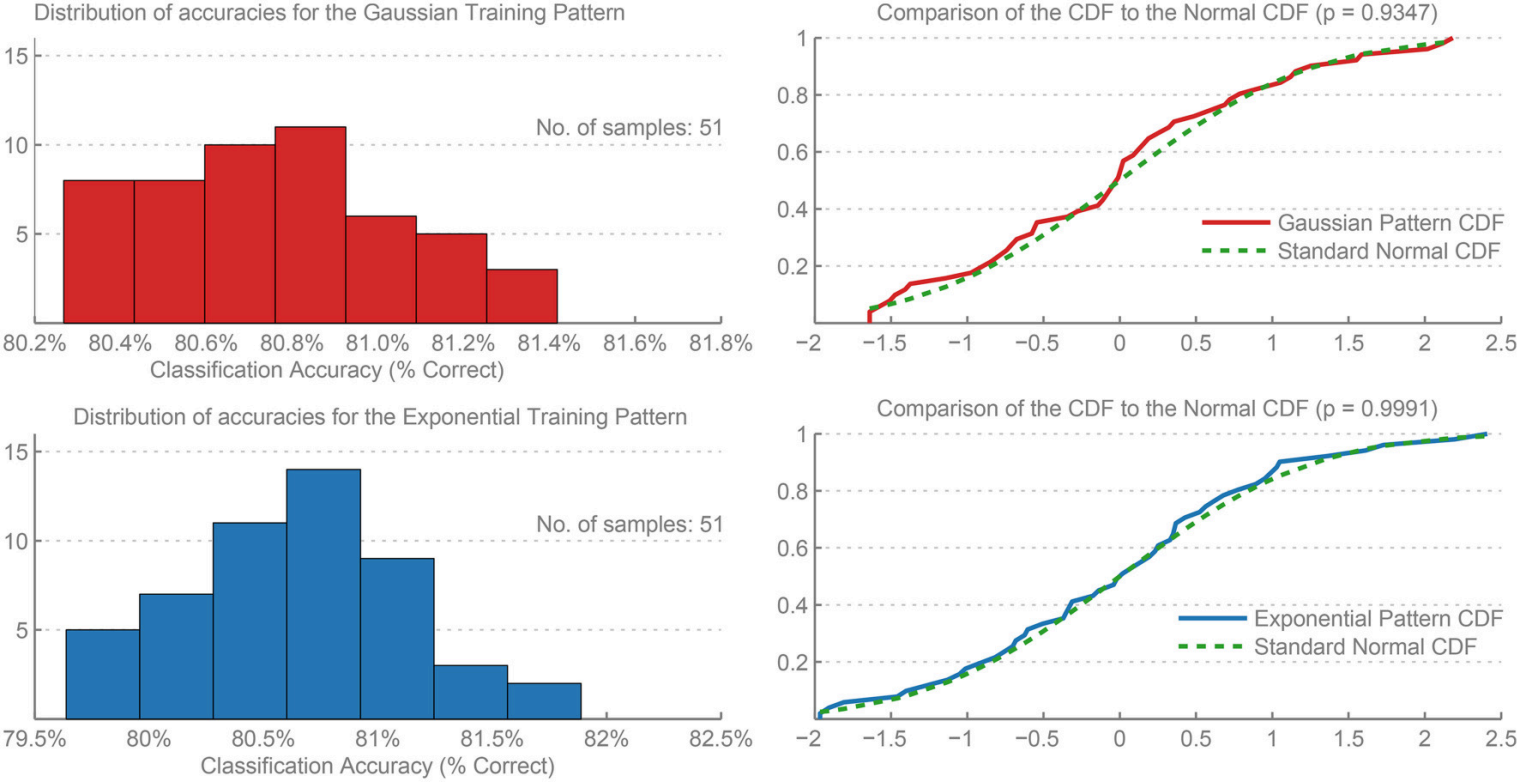
**Histograms of the distribution of errors and their Cumulative Distribution Functions (CDF) for the Gaussian and Exponential training patterns**. The results were calculated over 51 independent trials of each network. A comparison to the CDF of a Standard Normal distribution is included, and the *p*-value for a one-sample Kolmogorov-Smirnov test provided, demonstrating that both distributions are normally distributed.

Figure 5 shows the distribution of accuracies for the Exponential and Gaussian patterns for the 51 trials. A one-sample Kolmogorov-Smirnov test was used to test the normality of the distributions (Frank and Massey, 2011), and the null hypothesis was retained for both the Gaussian pattern (*p* = 0.9347) and the Exponential pattern (*p* = 0.9991).

Furthermore, applying the Lilliefor's composite goodness-of-fit test of composite normality (Lilliefors, 1967) (which itself is a specialized version of the Kolmogorov-Smirnov test) also retained the null hypothesis that the data are normally distributed (*p* > 0.5) for both patterns.

These results show that the output errors are normally distributed, and therefore are sufficiently represented by the mean and standard deviation of the accuracies or error rates.

### 3.3. Effect of training pattern on SKIM networks

**Figure 6:**
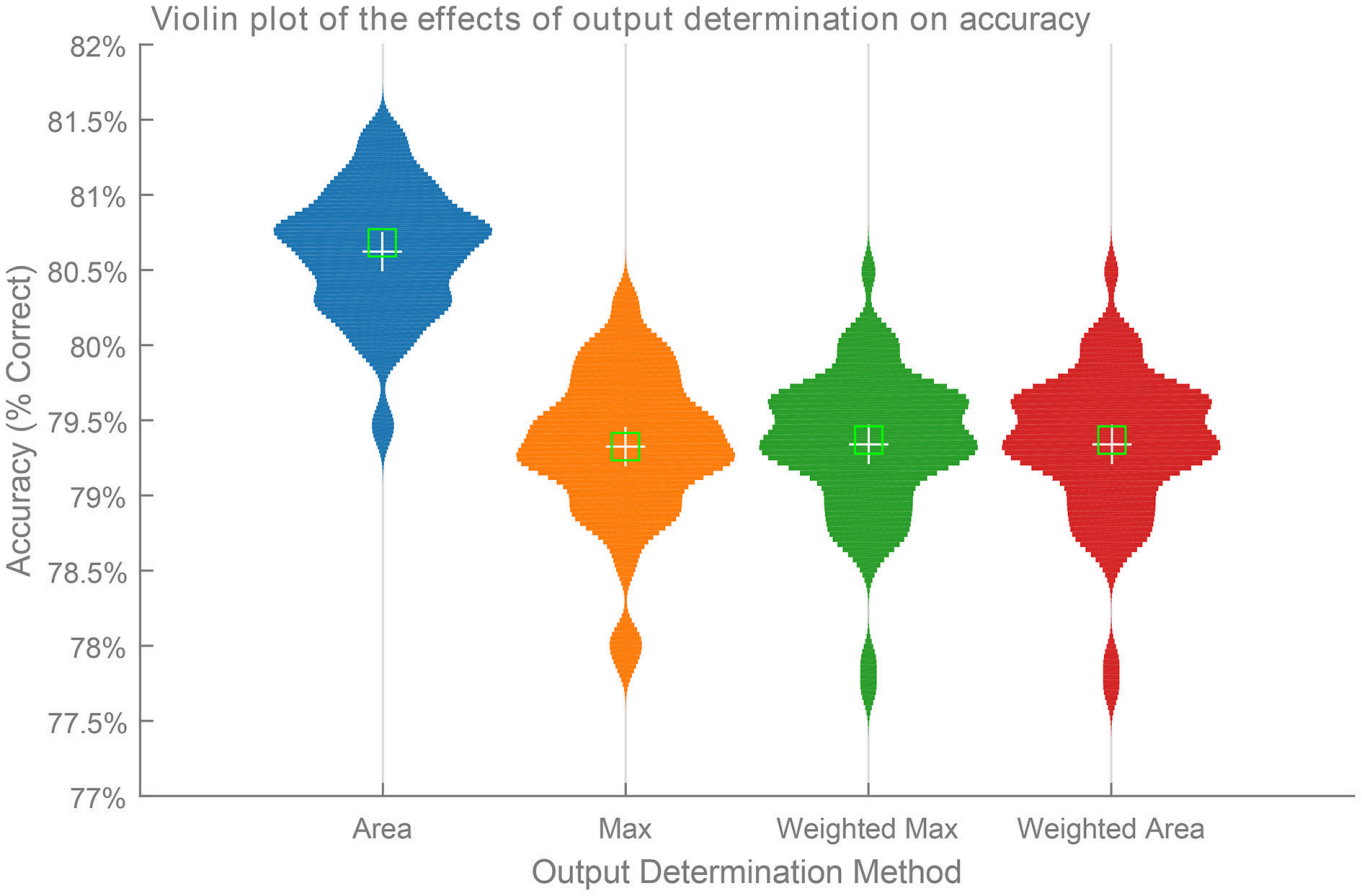
**Comparison of the effects of the four different output determination methods on classification accuracy with a Gaussian training pattern**. The figure shows the distribution of classification accuracies over 61 trials of a SKIM network with 1000 hidden layer neurons. Each network made use of the same random training order and hidden layer configuration, with only the random weights varying from trial to trial. The network made use of a Gaussian training pattern and uses the four different classification methods shown in Figure 3.

Figure 6 shows a comparison of the four output methods over 61 trials of a SKIM network consisting of 1000 hidden layer neurons and trained using a Gaussian training pattern with a μ of 10 and a σ of 5. The network structure and training order remained constant between each trial, with only the random input layer weights differing from trail to trial. Each output determination method ran on the same output for each trial, and calculated classification accuracy in terms of percentage of digits correctly identified.

It is immediately and apparently clear from the figure that the Area method produces the best result overall (*p* < 0.01). The performance of the other two methods did not show any statistically dominance at the 5% significance level.

The superior performance of the Area Method over the Weighted Area method is an interesting result, and shows that the learning mechanism makes use of the whole training pattern, and not simply the maximum value. As this method consistently produces the best results, all experiments henceforth report this result unless otherwise specified.

### 3.4. Effect of output determination method

The same random weights and hidden layer alpha functions were maintained across all trials, with only the training pattern varied across the tests. The classifiers all achieved accuracies consistent with the results expected for a network with 1000 hidden layer neurons. The experiments and tests (along with all others in this section) make use of an output pattern of 10 time-steps in length.

**Figure 7:**
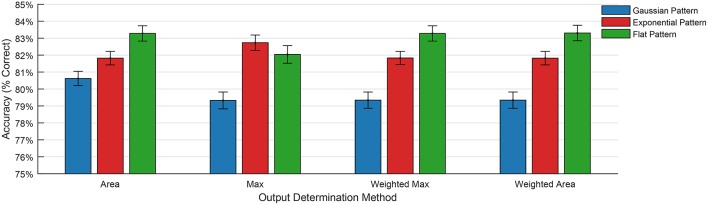
**Classification error when using different training patterns and output determination methods**.

Figure 7 shows the results of the training the three training patterns for the four output determination methods. The graph shows the mean error rate across all sixty trials for each training pattern. These results indicate that the Flat training pattern produces the best results in every case except for the Max determination method, and that the Gaussian method produces the worst result in every case.

Table 3 shows the mean accuracy and standard deviation resulting from the trials over sixty independent tests for all three training patterns. A difference was not observed in the performance of the area method under different training patterns (*p* = 0.091), but the performance of the Max method greatly improved (*p* < 0.01). The standard deviation in the results did not vary between the two output determination methods, and remained consistent.

**Table 3:** **Mean accuracies and standard deviation for the comparison between the Exponential, Flat, and Gaussian training patterns**.

	**Gaussian pattern**		**Exponential pattern**		**Flat pattern**	
	**Area (%)**	**Max (%)**	**Area (%)**	**Max (%)**	**Area (%)**	**Max (%)**
Mean	80.62	79.32	81.82	82.73	83.28	82.04
STD	0.42	0.49	0.45	0.40	0.45	0.51
Max	81.42	80.33	82.62	83.67	84.17	83.16
Min	79.41	77.88	81.12	81.82	82.36	81.06

*This table presents a comparison of the different training patterns under both the Area and Max output determination methods*.

Further investigation into these results shows that the initial discontinuity present in the flat and exponential patterns is the primary source of the performance improvement. The discontinuity produces a large and sudden spike in the error signal for the update stage. The Gaussian method produces a smooth error signal without any discontinuities, which has the effect of smoothing away the maximum peak. For this reason, the Max method is least effective when used with the Gaussian pattern.

Figure 7 also demonstrates an important link between training pattern and output determination method, and suggests that the choice of training pattern determines the optimal output determination method. The results show that the Area method is the best choice when using a Gaussian training pattern, and the Max method produces the best results when using an exponential training pattern. This makes sense when considering that the area under the Gaussian training pattern is larger, and thereby increases the total area under the output curve during recall. In a similar fashion, the sharp discontinuity, and resulting spike in the error signal, creates a more pronounced maximum value at the onset of the output pattern.

The flat output pattern benefits from the same effects from the sharp initial discontinuity, and also from the sharp negative error in the training signal resulting from the second discontinuity.

## 4. Discussion

This paper explores the use of the SKIM and OPIUM learning methods applied to event-based vision systems and large datasets and specifically in the realm of digit classification using an event-based camera. This work makes use of SKIM networks applied to the largest datasets to date, implementing the largest hidden layer sizes and simultaneously training the largest number of output neurons. The success of the classifiers built using these SKIM networks validates both the underlying SKIM algorithm and its applicability to event-based tasks such as the digit classification task presented in this work.

The classifiers presented in this work also achieve the highest accuracy on the N-MNIST dataset to date with an online training algorithm, and serves to further justify the use of SKIM as a means for learning in event-based systems.

This paper also explores the use of different training patterns and output determination methods on the N-MNIST dataset, and provides an analysis of the results, with Table 4 presenting a summary of the recommended output determination methods for the training patterns introduced in this work.

**Table 4:** **Recommended output determination methods for different training patterns**.

**Training pattern**	**Recommended output method**
Gaussian pattern	Area method
Exponential pattern	Max method
Flat pattern	Area or max method

One significant finding arising from the comparison of training patterns and output determination methods is that the Max method produces the best results when trained with an exponential pattern. This is important as the Max method is perhaps the simplest way of determining outputs in a system as it requires only a comparison operation. This is an important consideration as reductions in the complexity of the processing at the output neurons can greatly simplify implementation costs and reduce power consumption.

The SKIM algorithm itself poses certain challenges to a direct hardware implementation. The nature of the continuous elements and the transfer functions lend themselves to an analog implementation, and these results serve to prove the viability of such an approach. A mixed-signal implementation of the SKIM algorithm should produce a power-efficient and fully spike-based system, but requires that factors such as the size of the hidden layer and the nature of the transfer functions be fixed at design time.

This research serves to provide the first steps in validating the SKIM algorithm for real-world spike-based tasks, to provide insight into the performance of varying network sizes, and to demonstrate the scalability of the SKIM algorithm to real-world tasks.

Implementing the SKIM network using digital circuitry adds additional complications as the outputs from the hidden layer neurons require simulating regardless of the input activity. The finding that the Exponential pattern produces the best results for the Max Method is of particular significance as the Max Method of output determination offers the simplest and most direct hardware implementation.

Furthermore, the fact that the Exponential pattern starts at a maximum and then decays to zero opens up interesting methods for analytically calculating and comparing the outputs if the output weights are restricted to positive values only

It is also possible to implement the SKIM algorithm on existing large-scale neuromorphic hardware, such as SpiNNaker, leveraging the existing routing and processing infrastructure to implement the connectivity and transfer functions. In such a system, the internal timing limits dictate the maximum rate that the hidden layer nodes can produce values, thereby placing limits on the time resolution of the input spike patterns.

## Author contributions

GC performed the research and wrote the article in conjunction with GO. The vision aspects of the project were guided by RB and SL and the learning mechanisms were guided by JT and AV. All parties assisted in both the research and writing of this research.

### Conflict of interest statement

The authors declare that the research was conducted in the absence of any commercial or financial relationships that could be construed as a potential conflict of interest.
