# Supplementary material for: Skimming Digits: Neuromorphic Classification of Spike-Encoded Images
Source: Front Neurosci. 2016 Apr 28;10:184. doi: 10.3389/fnins.2016.00184 (PMC4848313; doi:10.3389/fnins.2016.00184)
Supplement: Supplementary file 1 [file Presentation1.ZIP › 5_NormalAnalysisGaussianExponential.pdf]

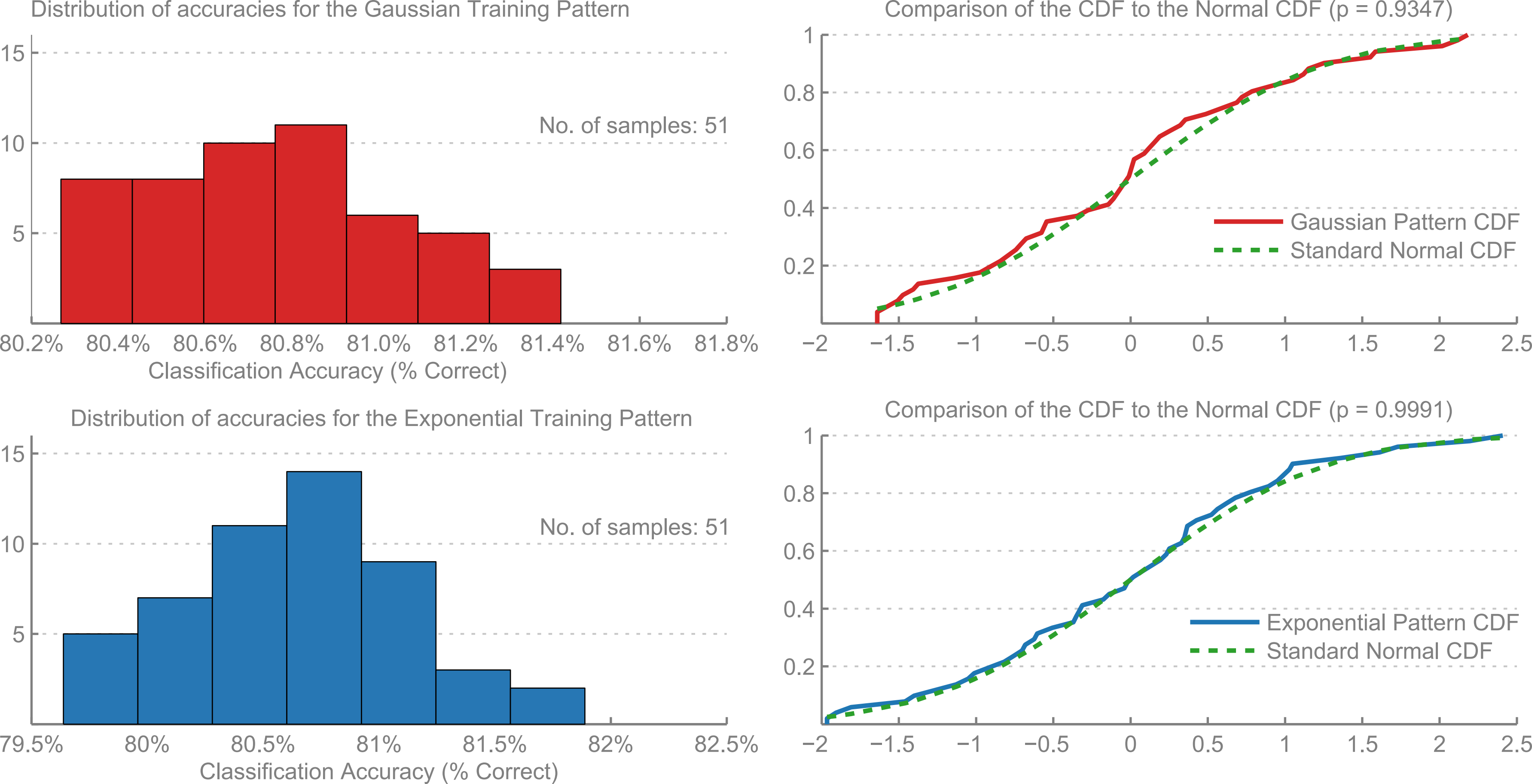
Distribution of accuracies for the Gaussian Training Pattern
No. of samples: 51
Classification Accuracy (% Correct)
80.2% 80.4% 80.6% 80.8% 81.0% 81.2% 81.4% 81.6% 81.8%
5 10 15
Comparison of the CDF to the Normal CDF (p = 0.9347)
Gaussian Pattern CDF
Standard Normal CDF
−2 −1.5 −1 −0.5 0 0.5 1 1.5 2 2.5
0.2 0.4 0.6 0.8 1
Distribution of accuracies for the Exponential Training Pattern
No. of samples: 51
Classification Accuracy (% Correct)
79.5% 80% 80.5% 81% 81.5% 82% 82.5%
5 10 15
Comparison of the CDF to the Normal CDF (p = 0.9991)
Exponential Pattern CDF
Standard Normal CDF
−2 −1.5 −1 −0.5 0 0.5 1 1.5 2 2.5
0.2 0.4 0.6 0.8 1
