# Supplementary figures and images for: Skimming Digits: Neuromorphic Classification of Spike-Encoded Images
Source: Front Neurosci. 2016 Apr 28;10:184. doi: 10.3389/fnins.2016.00184 (PMC4848313; doi:10.3389/fnins.2016.00184)

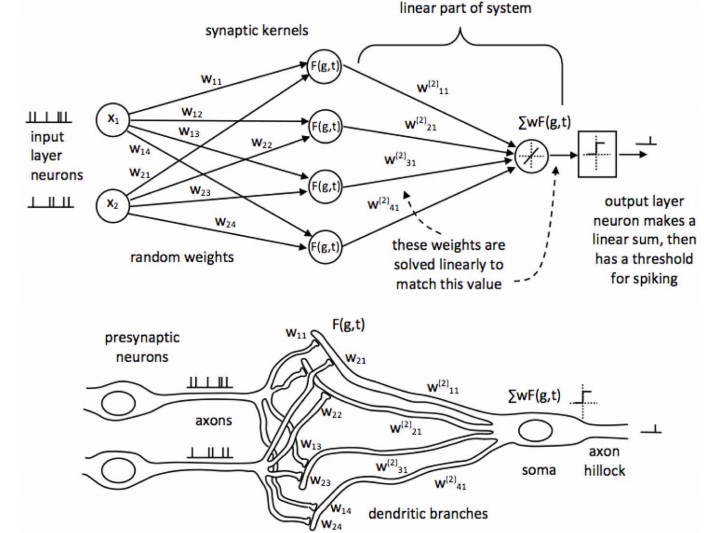

Supplement: Supplementary file 1 [file Presentation1.ZIP › 1_SKIMDiagram.jpg]

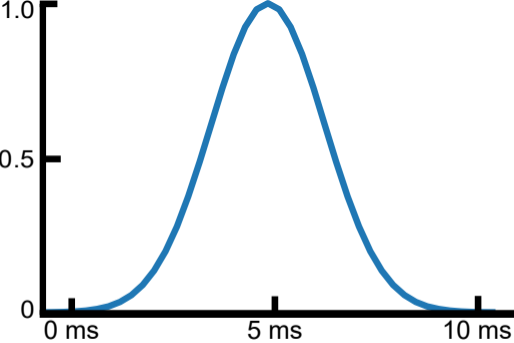

Gaussian Pattern

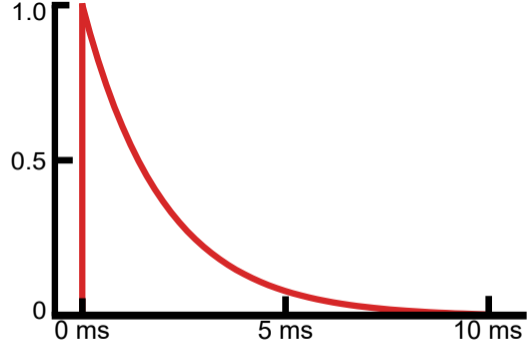

Exponential Pattern

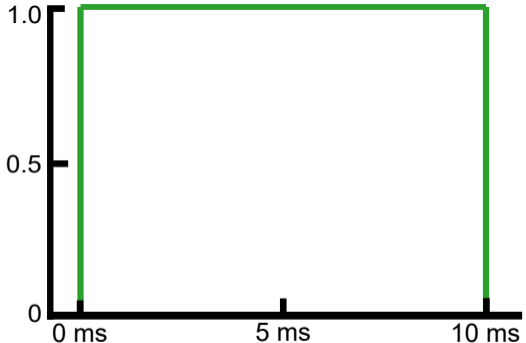

Flat Output Pattern

Supplement: Supplementary file 1 [file Presentation1.ZIP › 2_TrainingPatternTypes2.pdf]

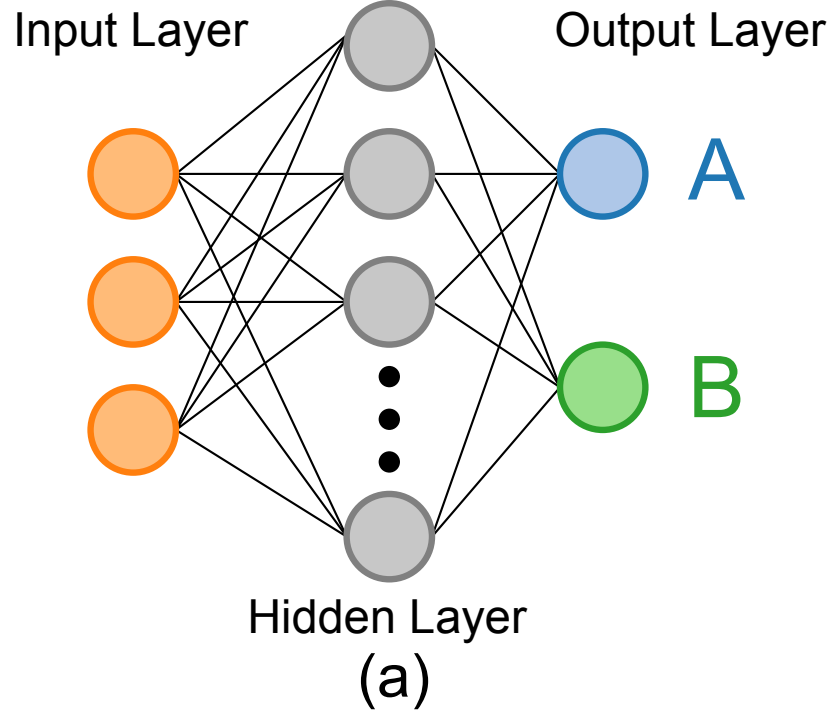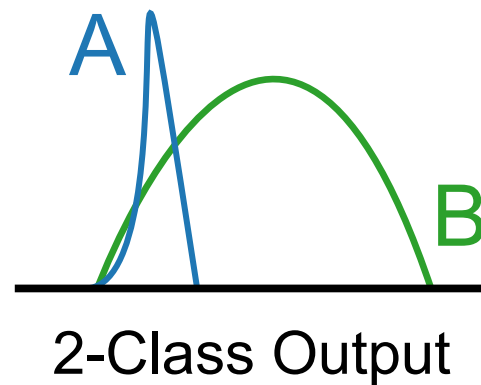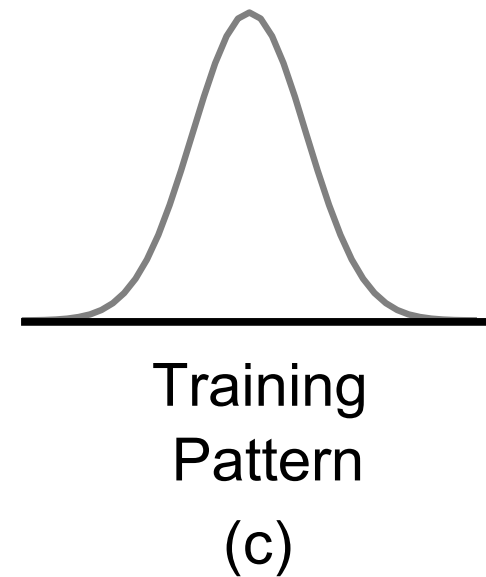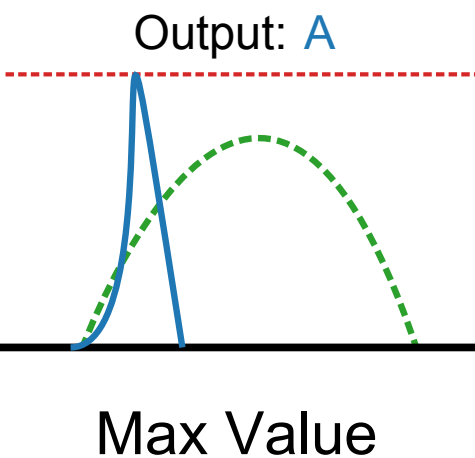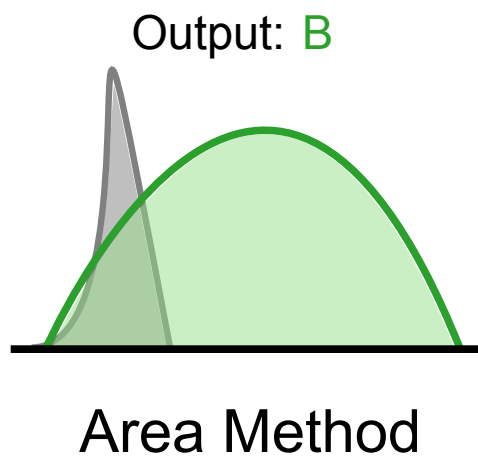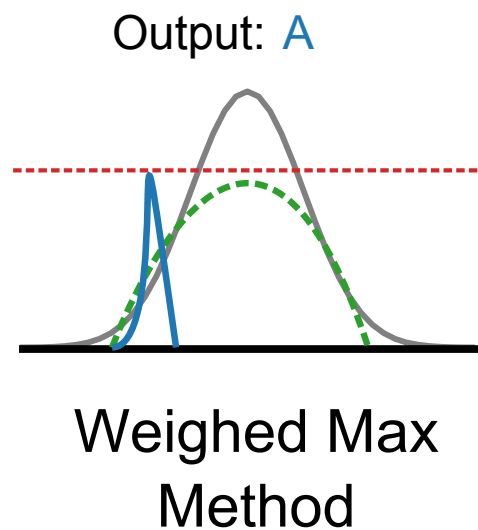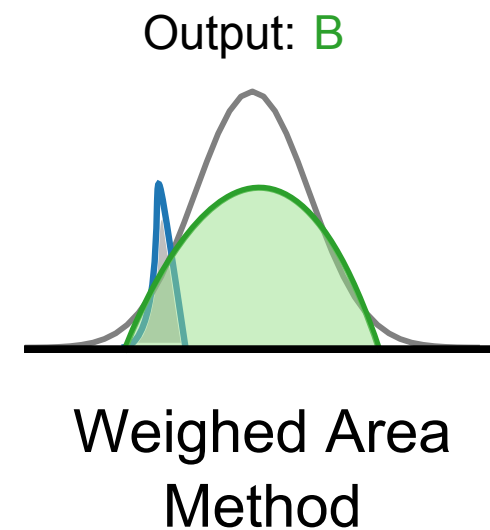

(d)

Supplement: Supplementary file 1 [file Presentation1.ZIP › 3_OutputDeterminationDiagram.pdf]

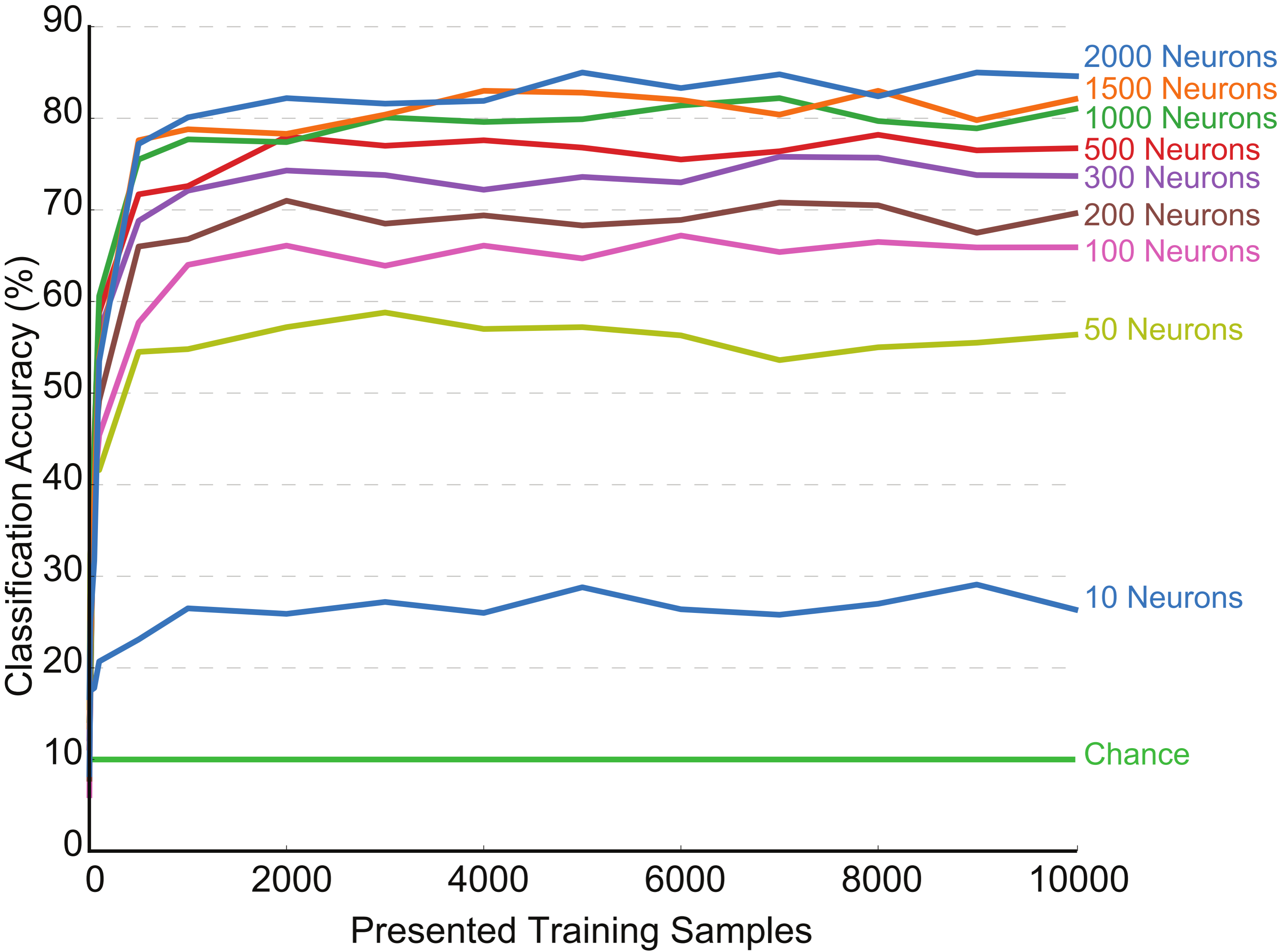

Supplement: Supplementary file 1 [file Presentation1.ZIP › 4_SKIM_MNIST2.pdf]

Violin plot of the effects of output determination on accuracy

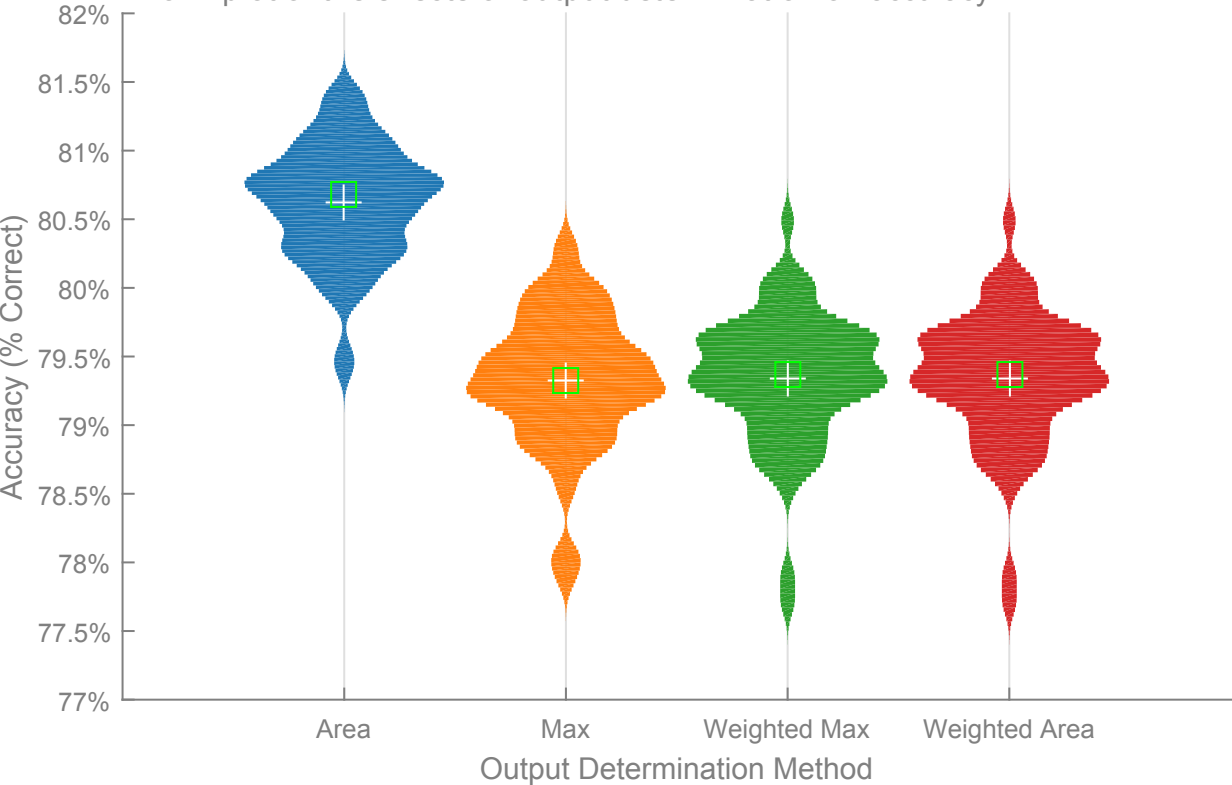

Supplement: Supplementary file 1 [file Presentation1.ZIP › 6_mnist-outputdetermination-methods.pdf]

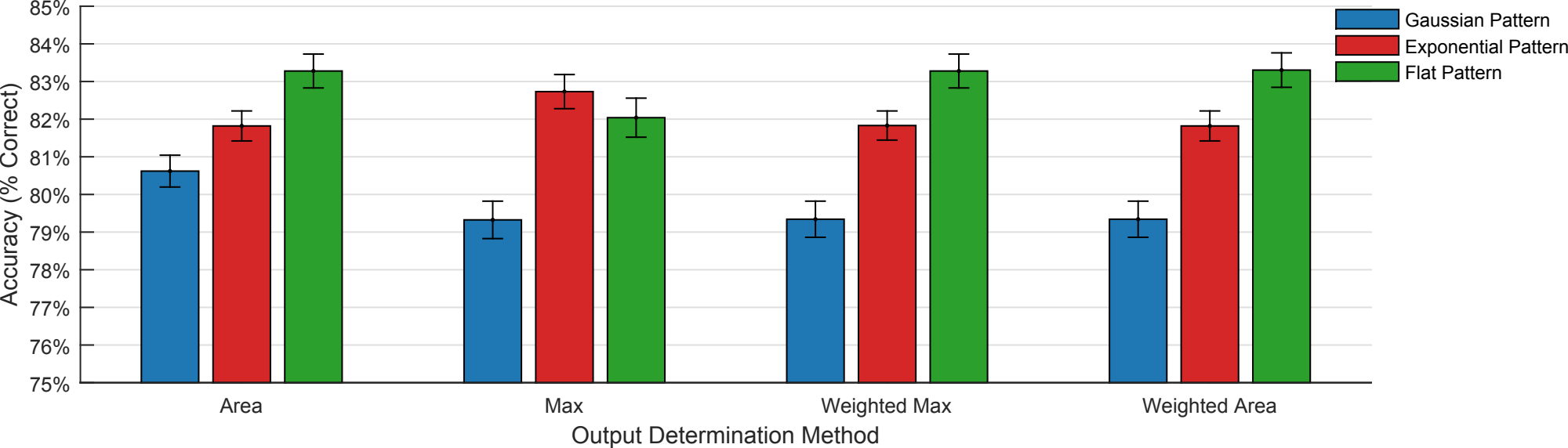

Supplement: Supplementary file 1 [file Presentation1.ZIP › 7_TrainingPatternComparisonBarChart2.pdf]
